# Anti-melanoma Differentiation-Associated Gene 5 (Anti-MDA5) Dermatomyositis-Associated Interstitial Lung Disease Complicated by Pneumomediastinum: A Case Report and Literature Review

**DOI:** 10.7759/cureus.64191

**Published:** 2024-07-09

**Authors:** Shivang Chaudhary, Amulya Balagani, Muhammad Zaheer

**Affiliations:** 1 Internal Medicine, Saint Louis University School of Medicine, St. Louis, USA; 2 Rheumatology, Saint Louis University School of Medicine, St. Louis, USA

**Keywords:** cyclophosphamide, pulse dose steroids, dermatomyositis, rapidly progressive interstitial lung disease, pneumomediastinum (pm), anti-mda5 amyopathic dermatomyositis

## Abstract

Anti-melanoma differentiation-associated gene 5 (anti-MDA5) dermatomyositis (DM) is a subset of amyopathic myositis and is associated with unique cutaneous manifestations and rapidly progressive interstitial lung disease (RP-ILD). A rare complication associated with high mortality is the occurrence of pneumomediastinum. We present a case of a 58-year-old female with anti-MDA5 DM-associated interstitial lung disease (ILD) complicated by pneumomediastinum. Treatment with pulse dose steroids and intravenous cyclophosphamide led to clinical improvement and resolution of the pneumomediastinum. Our case emphasizes the recognition of ILD-associated pneumomediastinum in patients with anti-MDA5 DM. Swift diagnosis and aggressive treatment are crucial due to the associated high mortality.

## Introduction

Clinically amyopathic dermatomyositis (CADM) is a subset of dermatomyositis (DM) that presents with the cutaneous manifestations of DM without muscle weakness [1]. In 2005, an antibody was discovered to be associated with CADM known as anti-melanoma differentiation associated with gene 5 (anti-MDA5) antibody [2]. Patients with anti-MDA5 DM have unique clinical manifestations such as cutaneous ulcerations, palmar papules, nonscarring alopecia, and panniculitis [3-5]. The most critical complication in these patients is the association with rapidly progressive interstitial lung disease (RP-ILD) with high morbidity and mortality. We present a case of a patient with anti-MDA5 DM-associated interstitial lung disease (ILD) complicated by pneumomediastinum.

## Case presentation

**Figure 1:**
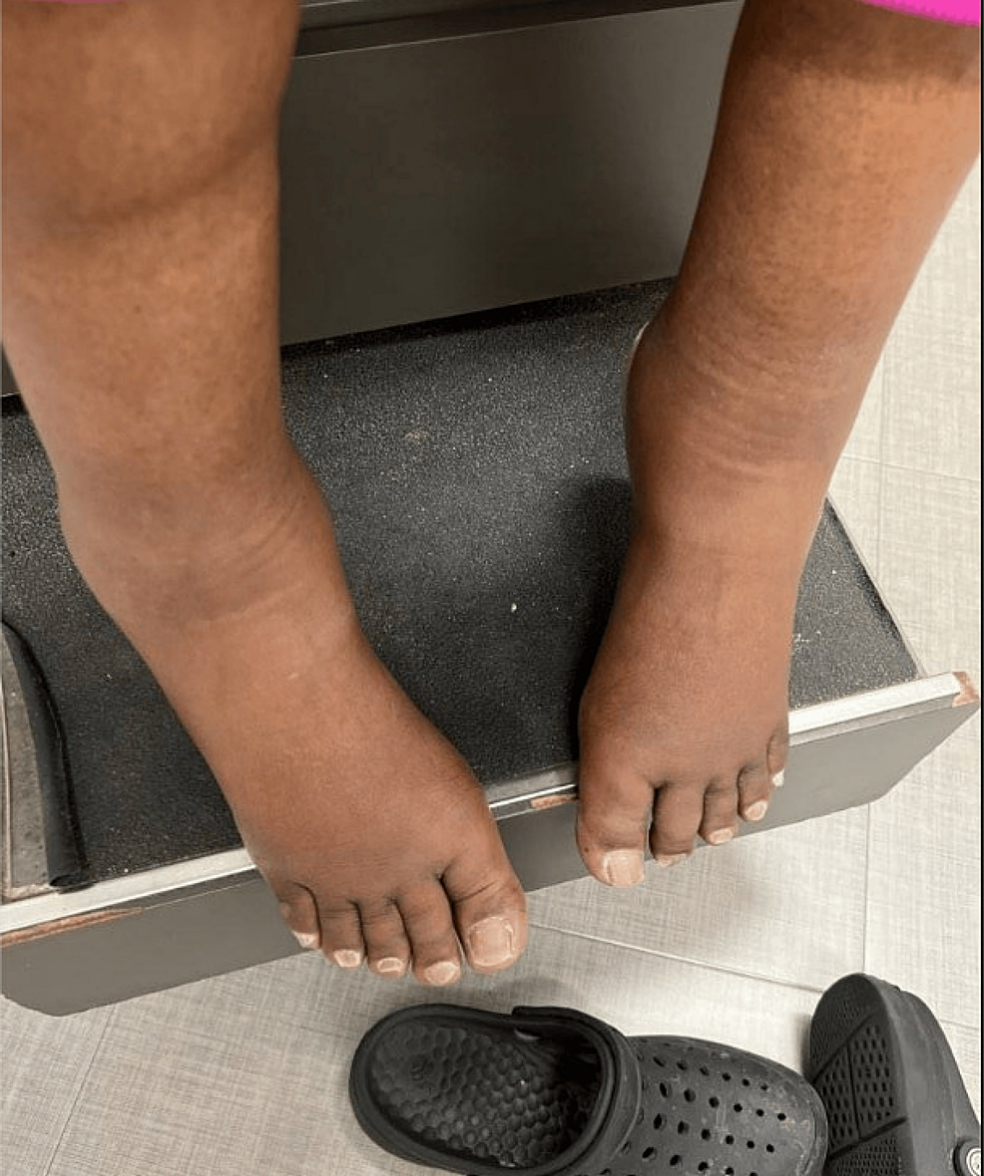
Image of patient’s feet showing significant ankle swelling

**Figure 2:**
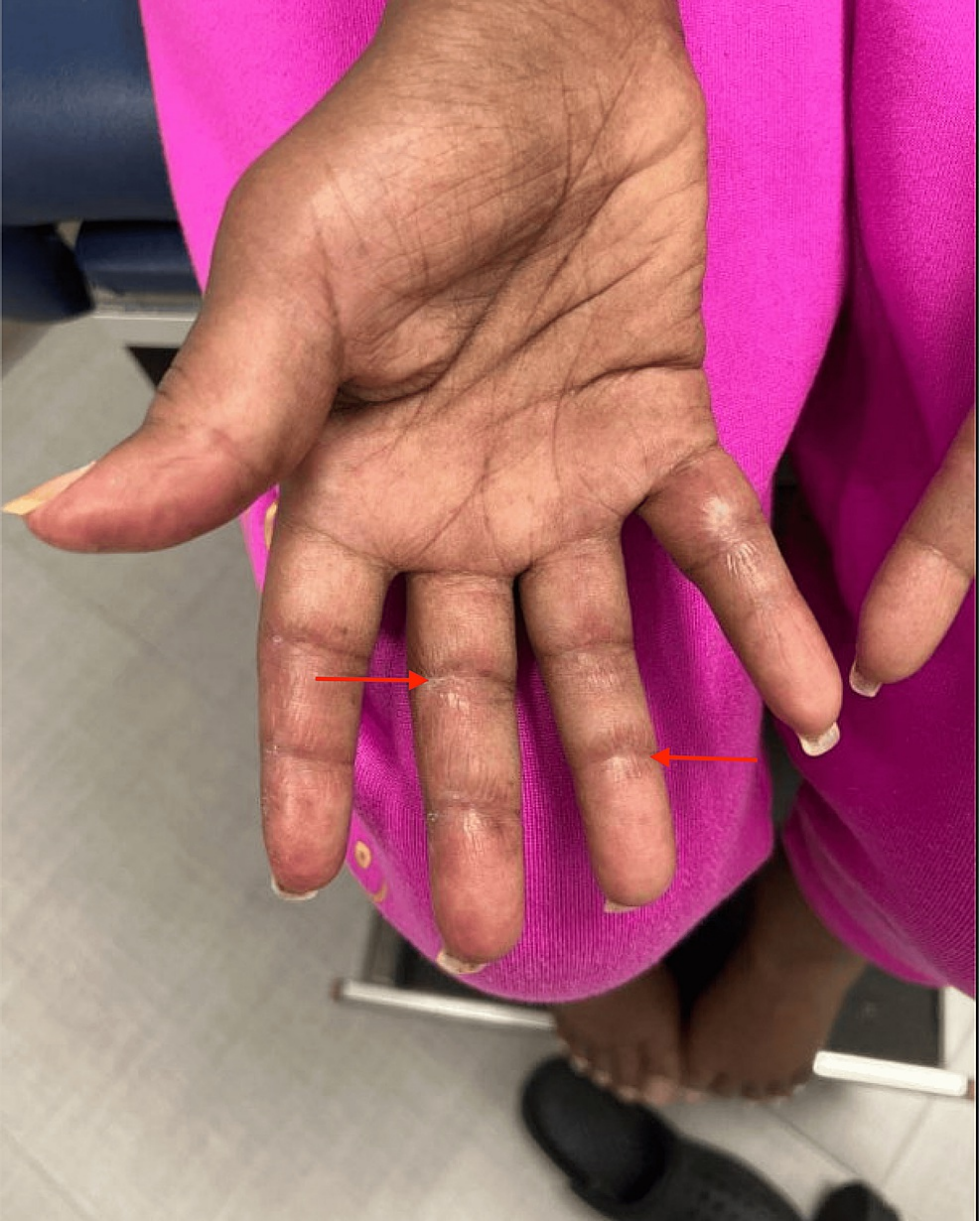
Image of patient's right hand showing healing cutaneous ulcerations (red arrows)

**Figure 3:**
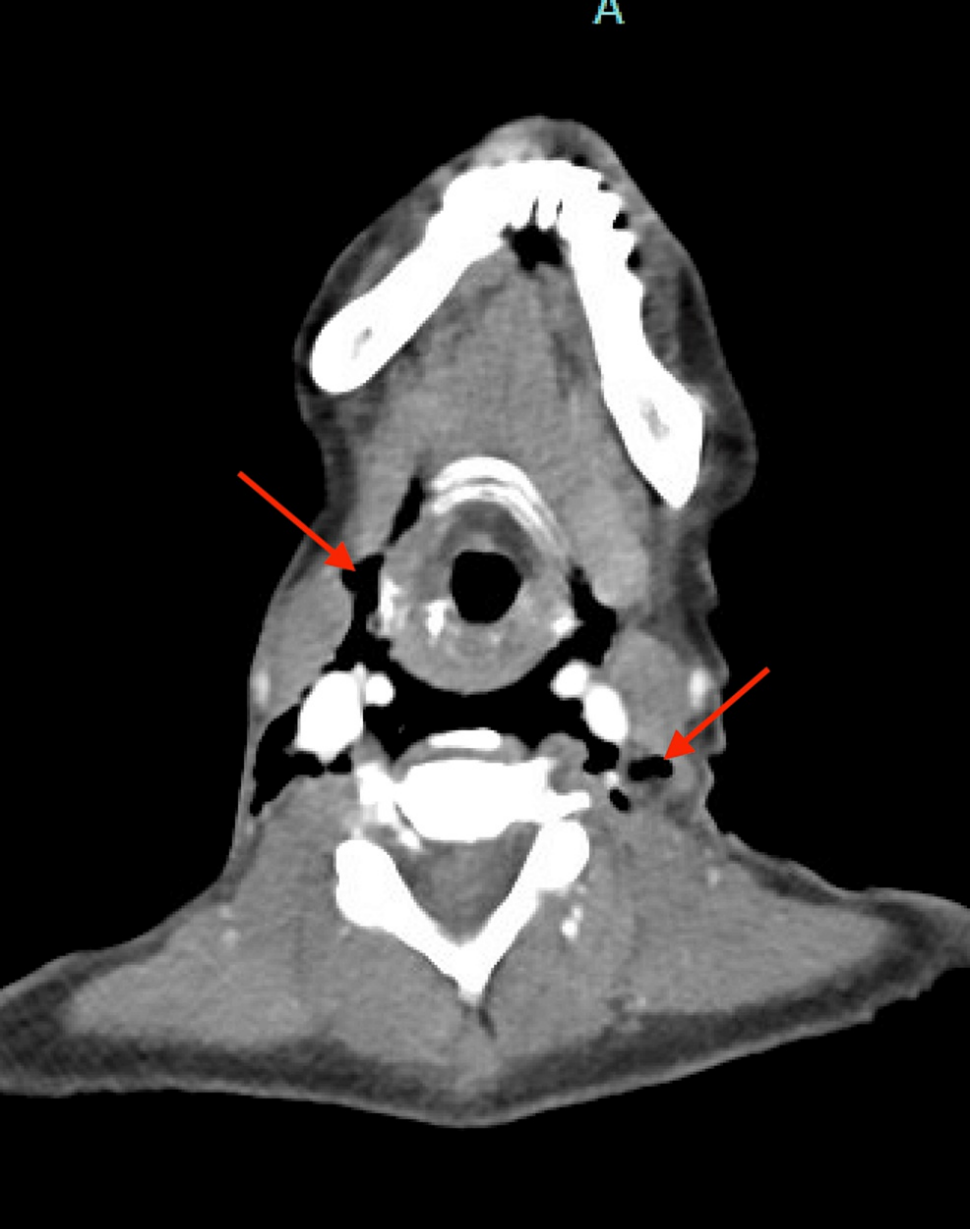
Axial slice of CT neck displaying air in the retropharyngeal space and deep neck spaces (red arrows)

**Figure 4:**
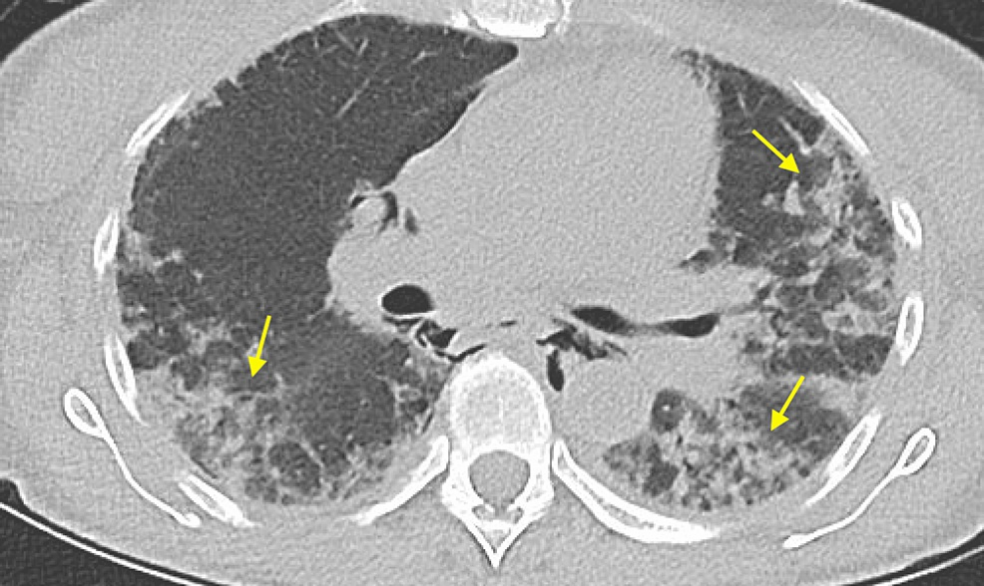
Axial view of CT chest displaying bilateral ground glass opacities and consolidation (yellow arrows)

**Figure 5:**
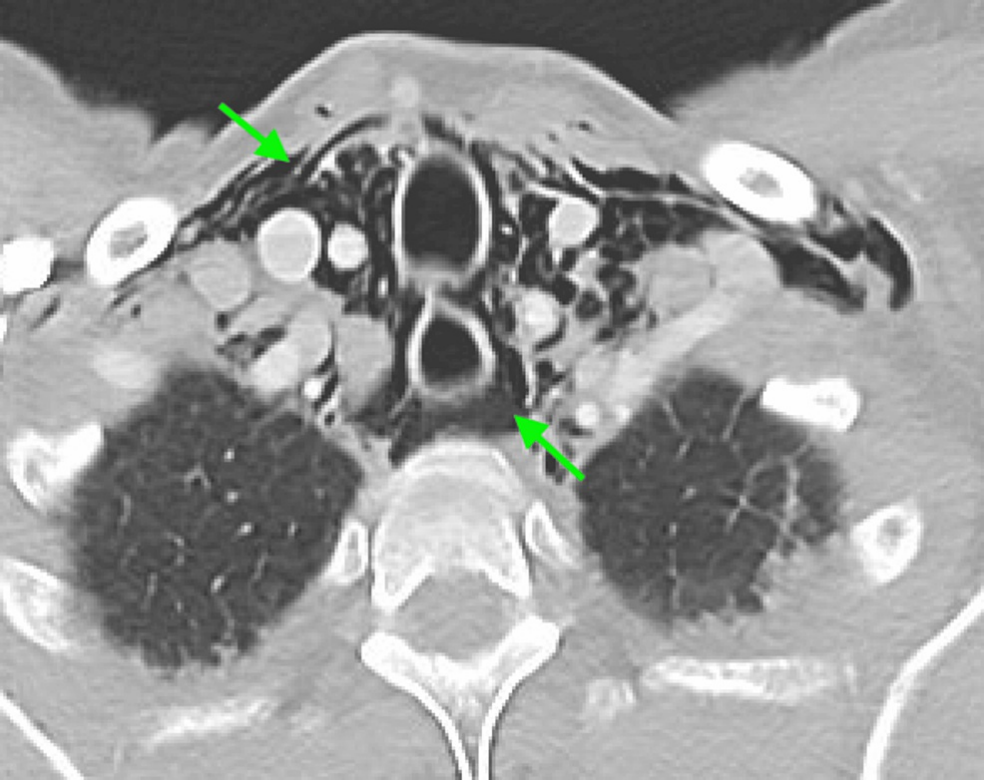
Axial slice of CT chest displaying extensive pneumomediastinum (green arrows)

A 58-year-old female presented to the hospital with a six-month history of pain and swelling of her fingers and ankles and approximately 100 pounds of weight loss in the last year. She had decreased exercise tolerance and difficulty functioning at work. Her past medical history was only significant for hypertension. She had never been a smoker and denied any other drug use. A physical exam revealed cutaneous ulcerations of her finger creases as well as swelling and tenderness of her small hand joints and ankles (Figures 1-2). She lacked hyperkeratosis and fissuring of her lateral fingers characteristic of mechanic’s hands. Crepitus was felt in the supraclavicular and anterior pectoral region and auscultation of her lungs revealed bibasilar inspiratory crackles. CT neck was obtained due to crepitus showing extensive subcutaneous emphysema involving the superficial and deep spaces of the neck and retropharyngeal space (Figure 3). CT chest revealed bilateral ground glass opacities in a nonspecific interstitial pneumonia pattern and pneumomediastinum most pronounced in the anterior and superior mediastinum (Figures 4-5). She had no evidence of muscle weakness on the exam. Laboratory data showed an unremarkable complete blood count and complete metabolic panel. She underwent extensive workup for ILD including antinuclear antibody (ANA) with extractable nuclear antigens profile, myositis panel, scleroderma comprehensive panel, and rheumatoid arthritis serologies. Results were significant for ANA titer of 1:320 via indirect immunofluorescent assay and high titer MDA5 antibody (Table 1) with the other serologies being negative. The erythrocyte sedimentation rate was elevated at >130 MM/HR. Other labs were significant for normal creatine kinase, aldolase, and C-reactive protein levels (Table 1). She was diagnosed with anti-MDA5 DM ILD complicated by pneumomediastinum based on characteristic cutaneous findings, normal muscle strength and muscle enzyme levels, and high titer MDA5 antibody. Pulmonary function testing showed moderate restrictive ventilatory limitation. She had an extensive negative workup for malignancies including CT scans of the abdomen and pelvis, and an ovarian ultrasound. Latent tuberculosis (TB) testing with interferon-gamma release assay was serially indeterminate, so the pulmonary team was consulted for bronchoscopy and bronchoalveolar lavage. However, the procedure was deemed to be high risk due to pneumomediastinum. CT imaging was not classic for pulmonary TB and the patient did not have risk factors for TB including no history of incarcerations, international travel, sick contacts, or occupational exposure. She had three negative sputum smears for acid-fast bacilli. The cardiothoracic surgery team recommended no surgical intervention for the pneumomediastinum, and based on a multidisciplinary team discussion, it was decided to treat her aggressively with immunosuppressive therapy for her underlying severe ILD. She was started on pulse dose intravenous (IV) methylprednisolone (1000 mg daily for three days) and IV cyclophosphamide (CYC) at a dose of 750 mg/m2 followed by oral prednisone 60 mg daily. She completed six monthly IV CYC infusions for induction of remission and prednisone was tapered down to 5 mg daily during this time. Mycophenolate mofetil was added for maintenance therapy at a dose of 500 mg twice daily and increased to 1500 mg twice daily. Repeated CT chest two and eight months after initiation of therapy showed resolved pneumomediastinum and no interval radiologic progression of interstitial lung disease. A follow-up physical exam showed no evidence of crepitus. The patient also improved clinically with increased exercise tolerance and was able to return to work.

**Table 1:** Laboratory values

Lab (Reference range)	Value
ANA-indirect immunofluorescence	Speckled 1:320 titer
Erythrocyte sedimentation rate (0-30 MM/HR)	>130 MM/HR
C-reactive protein levels (≤0.5mg/dL)	<0.5 mg/dL
Anti-MDA5 antibodies (<11 SI)	High titer (89 SI)
Creatine kinase (30-200 U/L)	56 U/L
Aldolase (1.2-7.6 U/L)	5.4 U/L

## Discussion

Anti-MDA5 DM was historically first reported in the Japanese population and prevalence is higher in Asians compared to Caucasians [2]. Anti-MDA5 DM-associated ILD is a rare disease with a poor prognosis despite treatment. The exact pathogenesis of why anti-MDA5 DM can lead to ILD and pneumomediastinum is unknown; however, current theories suggest ILD-related diffuse alveolar damage, DM-associated vasculopathy, and glucocorticoid use. For DM-associated vasculopathy, it is proposed that alveoli are damaged due to ischemia caused by type 1 interferon (IFN) signaling. IFN-1 triggers the release of endothelin, which acts as a vasoconstrictor causing focal ischemia. Lastly, it is proposed that the use of glucocorticoids weakens the pulmonary interstitium through its effects on protein metabolism; however, studies do not show pulse dose steroid therapy as a predictor of developing pneumomediastinum in connective tissue diseases [6].

There is a paucity of randomized controlled studies guiding treatment for anti-MDA5 DM ILD. Based on the literature review, a combination immunosuppressive therapy is the standard of care in the form of glucocorticoids with CYC or triple therapy with the addition of calcineurin inhibitors or mycophenolate mofetil [7]. There is also data to suggest the role of Janus kinase inhibitors like tofacitinib [8] and monoclonal antibodies such as rituximab [9]; however, larger randomized controlled studies need to be done. Another case report demonstrated successful management using high-dose glucocorticoids followed by cyclosporine A and hydroxychloroquine [10]. The data suggests a survival benefit with upfront combination therapy compared to step-up therapy [1,11,12]. Our patient responded well to treatment with high-dose glucocorticoids in combination with IV CYC. However, outcomes often remain poor despite aggressive treatment using similar approaches in multiple cases [13].

Patients with RP-ILD in anti-MDA5 DM have an increased 90-day mortality and six-month mortality rate as high as 50 percent despite aggressive treatment with glucocorticoids and CYC [1].

## Conclusions

In conclusion, ILD with pneumomediastinum is a serious complication associated with high mortality in patients with anti-MDA5 DM. As seen in our case, prompt identification of this severe entity and aggressive treatment are critical due to the associated high mortality. We demonstrate the successful use of high-dose glucocorticoids combined with IV CYC to control the progression of the ILD. Further studies are needed for a better understanding of the pathogenesis and to develop standardized and more effective therapeutics.
